# Usability of Telemedicine Mobile Applications during COVID-19 in Saudi Arabia: A Heuristic Evaluation of Patient User Interfaces

**DOI:** 10.3390/healthcare9111574

**Published:** 2021-11-18

**Authors:** Raniah N. Aldekhyyel, Jwaher A. Almulhem, Samar Binkheder

**Affiliations:** Medical Informatics and e-Learning Unit, Medical Education Department, College of Medicine, King Saud University, Riyadh 11451, Saudi Arabia; jalmulhem@ksu.edu.sa (J.A.A.); sbinkheder@ksu.edu.sa (S.B.)

**Keywords:** telemedicine, mHealth, heuristic evaluation, usability, Saudi Arabia, COVID-19

## Abstract

The coronavirus disease 2019 (COVID-19) pandemic has impacted the use of telemedicine application (apps), which has seen an uprise. This study evaluated the usability of the user interface design of telemedicine apps deployed during the COVID-19 pandemic in Saudi Arabia. It also explored changes to the apps’ usability based on the pandemic timeline. Methods: We screened ten mHealth apps published by the National Digital Transformation Unit and selected three telemedicine apps: (1) governmental “Seha”^®^ app, (2) stand-alone “Cura”^®^ app, and (3) private “Dr. Sulaiman Alhabib”^®^app. We conducted the evaluations in April 2020 and in June 2021 by identifying positive app features, using Nielsen’s ten usability heuristics with a five-point severity rating scale, and documenting redesign recommendations. Results: We identified 54 user interface usability issues during both evaluation periods: 18 issues in “Seha” 14 issues in “Cura”, and 22 issues in “Dr. Sulaiman Alhabib”. The two most heuristic items violated in “Seha”, were “user control and freedom” and “recognition rather than recall”. In “Cura”, the three most heuristic items violated were “consistency and adherence to standards”, “esthetic and minimalist design”, and “help and documentation” In “Dr. Sulaiman Alhabib” the most heuristic item violated was “error prevention”. Ten out of the thirty usability issues identified from our first evaluation were no longer identified during our second evaluation. Conclusions: our findings indicate that all three apps have a room for improving their user interface designs to improve the overall user experience and to ensure the continuity of these services beyond the pandemic.

## 1. Introduction

The coronavirus disease 2019 (COVID-19) pandemic has negatively impacted the world on different dimensions. The virus has spread rapidly with more than 196 million confirmed cases as of the 1st of August 2021 [1]. The threat of an imminent surge of COVID-19 patients drove healthcare organizations to act quickly to develop and deploy mobile health technologies [2,3], with telemedicine solutions in particular seeing an uprise [2,4,5]. Like other countries, healthcare organizations in Saudi Arabia responded to the pandemic by creating strategies to control the spread of disease, including the use of mHealth apps to provide telemedicine care for their patients [6,7]. While many studies have shown the benefits of telemedicine apps on patients and providers [2], the usability of these apps needs to be addressed more fully [8].

Ensuring excellent usability is at the core of patient engagement [9]. Given the rapid increase in telemedicine apps during the pandemic and insufficient usability assessments, the potential impacts on user engagement and experience are not clear but are substantial [10]. Performing standardized usability assessments designed to capture the user’s experience with telemedicine apps is critical in ensuring a positive user experience. Usability is defined by the International Organization for Standardization (ISO) as “the extent to which the product can be used by specified users to achieve specified goals with effectiveness, efficiency, and satisfaction in a specified context of use.” [11]. Usability is considered as a vital measure that captures users’ experience and helps inform the design of mHealth apps [12]. Researchers can use several methods to evaluate usability, including the heuristic evaluation method, involving several experts examining the system’s interface design [13]. Heuristic evaluation has been used extensively by different researchers [14,15,16,17,18] due to its low cost, ease of use, and the involvement of a small number of experts [19,20].

Saudi Arabia has many advances in digital healthcare, with specific strategic plans put in place for the advancement of healthcare using information technology [21]. Changes in insurance policies announced by the Saudi Council of Cooperative Health Insurance [22] during the pandemic indicating that telemedicine services would be covered by insurance companies influenced the rapid deployment of telemedicine services. While the effectiveness of telemedicine care has been published in the literature [23], with specific studies focusing on telemedicine user satisfaction during the pandemic [24,25,26,27], little is known about the ease and usability of telemedicine apps [8].

In this study our goal was to complete a heuristic evaluation to assess the usability of telemedicine apps, deployed in Saudi Arabia during the pandemic. We conducted the usability evaluation using Jakob Nielsen’s 10 usability heuristics for interface design [13]. We also explored changes to the usability of apps based on the pandemic timeline through conducting the evaluation during two different time periods.

## 2. Materials and Methods

We followed three phases in our study: Phase I was selecting telemedicine apps, Phase II was conducting the heuristic evaluation during two different time periods, and Phase III was data analysis. We conducted the first evaluation one month after announcing the first COVID-19 case in Saudi Arabia [28], and the country’s lockdown during April 2020, while the second evaluation was 14 months after our first evaluation (June 2021). We followed the same heuristic evaluation process during both evaluation periods.

### 2.1. Phase I. Telemedicine Apps Selection

In line with the government lockdown measures, the Saudi National Digital Transformation (NDT) Unit [29] during the time of our study published a document outlining a total of 10 mHealth apps (Appendix A, Table A1). On the 11th of April 2020, we independently reviewed the document and selected apps that met the criteria of a telemedicine mobile app, based on the definition of “telemedicine” as outlined in the National Saudi Telemedicine Policy: “mobile applications that provide remote interaction between a patient and a healthcare provider delivered through video, and/or audio, and/or picture, and/or text, and/or data” [30]. Any mHealth app, which did not include a telemedicine feature, such as apps developed for medication delivery, medical encyclopedias, or patient portals were excluded from our evaluation The three apps we selected covered three main types of telemedicine services; (1) the governmental app “Seha”^®^ [7], (2) the stand-alone private app “Cura”^®^ [31], and (3) a private app called “Dr. Sulaiman Alhabib”^®^, which is a paid telemedicine service provided by a private hospital [32].

#### Apps Description

“Seha” app provides free telemedicine consultation services for all citizens and residents. Users are required to register in the app using their mobile number. Once registration is confirmed through a text message sent to the user’s mobile, users can request for a consultation with a Ministry of Health’s physician up to three times per month. The app is not linked to a certain hospital/clinic nor to a specific unified patient medical record number. The app also includes an artificial intelligence technology feature in the form of an automatic health assessment tool.

“Cura” app is a stand-alone telemedicine app providing a paid consultation service to its users. The app offers on-demand consultations with general practitioners, specialists, and consultants. Users can choose a consultation with a specific physician from viewing a list of available physicians. The app also offers different wellness program packages. Like “Seha”, users register once using their mobile number and receive a confirmation through a text message. Consultations are offered with a fee that users are required to pay in advance. The app is not linked to a certain hospital/clinic or a specific unified patient medical record number.

“Dr. Sulaiman Alhabib” app is developed by Dr. Sulaiman Al Habib Medical Group; a private hospital with over 10 branches in Saudi Arabia. The app provides a variety of services for the hospitals’ patients and is integrated with their medical record system. The app provides a wide range of services for patients. Telemedicine consultation feature was added during the early months COVID-19 pandemic in 2020. The number of consultations offered to its users is based on their specific insurance coverage.

### 2.2. Phase II. Evaluation Procedure

To conduct the usability evaluation, we used Jakob Nielsen’s 10 usability heuristics for interface design [13,33] due to their widespread use [14,15,16,17,18]. After we performed an unstructured qualitative overview of the three apps, we designed an online form using google forms [34], which contained two sections: (1) features of the apps using a yes/no nominal scale, and (2) Nielsen’s ten usability heuristics with a 5-point severity rating scale [35] (Appendix B).

Given that we have no affiliation with the organizations, which developed the apps included in our study, and our background in health informatics and experience in usability testing and evaluation methodologies, we conducted the evaluation ourselves. Before each evaluation, we briefly discussed the heuristics and the severity classification to ensure that we followed a standardized evaluation process. Each of us then installed the three apps on our personal mobile phones (iPhone 11) and registered to access the apps. Using the standardized online form, we independently reviewed the apps and completed a real time teleconsultation with a physician to identify compliance with the heuristics. We completed separate forms to identify the apps’ features, record issues related to the heuristics, provide descriptions, and assign the severity ratings, and record the location of the issues.

### 2.3. Phase III. Data Analysis

Following the evaluation, we compiled the forms into a single form, and together we discussed our findings, generated consensus ratings, and provided redesign recommendations. We calculated frequencies and percentages for the usability issues and assigned the location of the issues to one of the following categories: (1) registration, (2) log in, (3) orientation on how to use the app, (4) initiating a consultation, (5) waiting for physician (6) during consultation, and (7) end of consultation. We identified the categories based on the steps users would follow to complete a consultation with a physician through the apps.

After completing both evaluations, we further analyzed our findings by examining the usability issues resulting from the first evaluation to check if they were still an issue in our second evaluation or were they resolved.

To avoid bias, we followed the recommendations outlined by McDonagh et. al in selecting studies for review [36], specifically: (1) defining an inclusion and exclusion criteria, and (2) applying dual review during the selection and evaluation phases—having two evaluators independently assess mhealth apps for inclusion and evaluate the apps using Nielsen’s ten usability heuristics. The same evaluators conducted both evaluations and none were affiliated with the organizations responsible for developing the apps.

## 3. Results

Table 1 shows an overview of the features of the three apps.

A summary of the usability issues identified in “Seha”, “Cura”, and “Dr. Sulaiman Alhabib” apps during the two evaluation periods, with the location of issues, severity rating, and redesign recommendations are presented in Table A2, Table A3 and Table A4 respectively (Appendix C). In total, we identified 54 user interface usability issues during both evaluation periods: 18 issues in “Seha” app (9 from the first and 9 from the second), 14 issues in “Cura” app (9 from the first and 5 from the second), and 22 issues in “Dr. Sulaiman Alhabib” app (12 from the first and 10 from the second). In “Seha” app, the two most heuristic items violated were “user control and freedom” and “recognition rather than recall”, with three unique usability issues identified in each. We found no issues under the “recognition diagnosis, and recovery from errors” heuristic. In “Cura” app the three most heuristic items violated were “consistency and adherence to standards”, “esthetic and minimalist design”, and “help and documentation”, with three unique usability issues identified in each. We found no issues under the two heuristics: “visibility of system status” and “recognition diagnosis, and recovery from errors”. In “Dr. Sulaiman Alhabib” app the most heuristic item violated was “error prevention”, with four unique usability issues identified, followed by “user control and freedom”, and help and documentation”, with three unique usability issues identified in each. The “flexibility and efficiency of use” heuristic item among all apps did not include accelerators or an ability to tailor frequent actions based on inexperienced and experienced users, therefore we considered this item not applicable in our evaluation.

Based on the location of issues among the three apps, we found the most usability issues were during the “consultation initiation” (*n* = 21), followed by “orientation” (*n* = 9), “during consultation” (*n* = 7), “registration” (*n* = 5), and “login” (*n* = 5). The least number of issues were categorized as “waiting for physician” (*n* = 4), and “end of consultation (*n* = 3). Notably, results of our first evaluation showed two location categories: “orientation” and “consultation initiation” related to the nine usability issues identified in “Cura” app. The only five usability issues categorized as “registration” were found in “Dr. Sulaiman Alhabib” app, and the only four usability issues categorized as “waiting for physician” were identified in “Seha” app.

**Figure 1:**
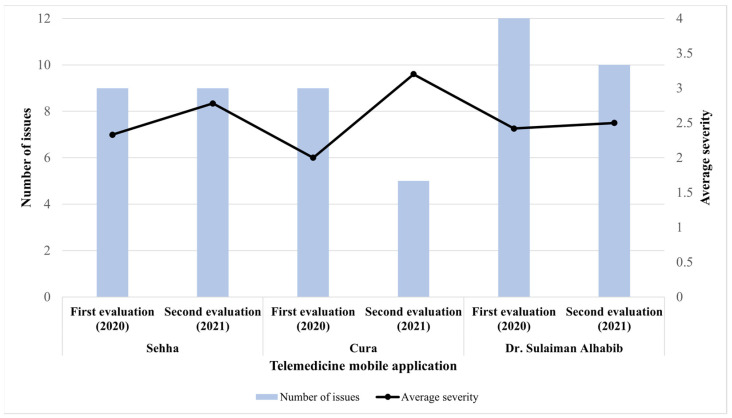
Frequency and average severity ratings by evaluation period among the three apps.

When we compared between the two evaluation periods, the numbers of usability issues in “Seha” app were similar in both evaluations, however the average severity rating was slightly higher in the second evaluation. In “Cura” app, the number of usability issues in the second evaluation was lower while the average severity rating was considerably higher in the second evaluation compared to the first evaluation. Average severity ratings for the “Dr. Sulaiman Alhabib” app was slightly changed between both evaluations while the number of usability issues was higher in the first evaluation in contrast to the second evaluation (Figure 1).

**Figure 2:**
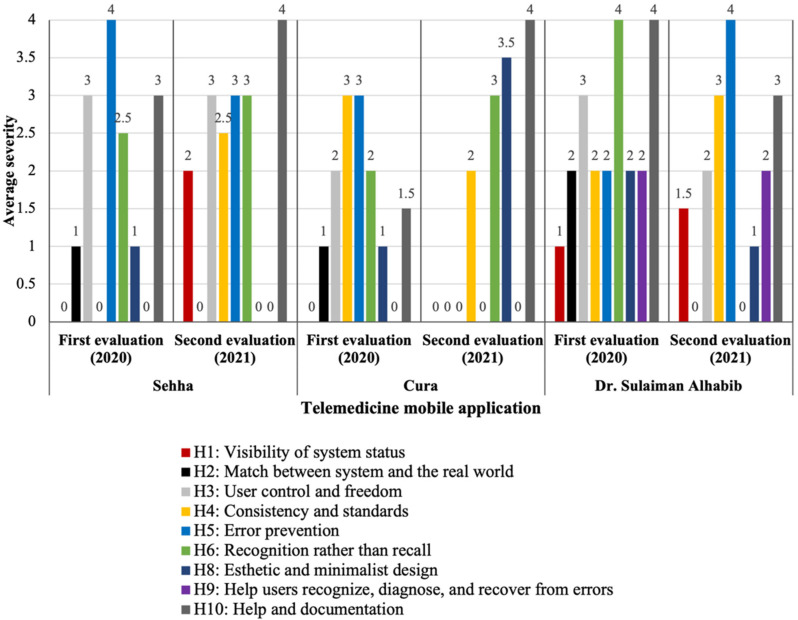
Average severity ratings by heuristic items among the three apps. H7: Flexibility and efficiency of use is not displayed in the chart.

The average severity ratings based on the heuristics in “Seha” app, showed two catastrophic issues: “error prevention” (identified from the first evaluation), and “help and documentation” (identified from the second evaluation). In the “Cura” app, issues related to both “consistency and standards” and “error prevention” items were rated as major issues in the first evaluation. Notably, five out of ten heuristic items did not involve any usability issues in the second evaluation. In “Dr. Sulaiman Alhabib” app, issues related to “recognition rather than recall” and “help and documentation” were rated as catastrophic in the first evaluation and issues related to “error prevention” were rated as catastrophic in the second evaluation (Figure 2).

Our first evaluation resulted in the discovery of 30 user interface usability issues among the three apps, with 10 of these issues no longer identified from our second evaluation. Three out of nine issues in both “Seha” and “Cura” apps were resolved, while four out of 12 issues were resolved in “Dr. Sulaiman Alhabib” app (Table A2, Table A3 and Table A4 Appendix C).

## 4. Discussion

Several telemedicine apps have been developed in Saudi Arabia ranging from free to paid services in response to the pandemic. With the increased availability of these apps, it is essential to measure the apps’ usability from a user’s perspective, to ensure continuity of these services beyond the pandemic. Our study was conducted to explore the usability issues related to three telemedicine apps used in Saudi Arabia during the pandemic, using Nielsen’s 10 heuristics. We performed two evaluations during two time periods to explore any changes to the usability of apps based on the pandemic timeline. We found that following a standardized approach in identifying the features of the telemedicine apps along with conducting the heuristic evaluation was a feasible and efficient method to evaluate the apps’ user interfaces. This method helped highlight positive features as well as classify usability issues, which may potentially assist the apps’ developers in resolving issues in future updates. We also used a standardized severity rating score for each issue we identified based on the 10 heuristics items. The rating helped highlight the significant usability issues and prioritize them to allocate possible resources in overcoming these issues [13,14]. Our evaluation also suggested possible redesign solutions, which if implemented can potentially enhance the overall user experience.

When developing telemedicine apps, healthcare organizations providing telemedicine services in Saudi Arabia must be aware of the current governing regulations [37,38,39,40], and accreditation bodies [41]. During the pandemic many efforts have been made by these organizations to develop and update their regulations to serve as a guide for healthcare organizations and developers. A national online training course for healthcare providers has also recently been launched by the Saudi Commission for Health Specialties to ensure a standardized approach in providing telemedicine care [42]. Utilizing these resources would ensure a high standard of telemedicine care and an overall positive user experience.

Beyond the results of our usability evaluation, our study demonstrated four key findings. First, the “Dr. Sulaiman Alhabib” app was the only app in our study linked to a hospital medical record system. “Seha” and “Cura” apps, which lacked integration with a medical record system may potentially affect the overall patient care experience since the medical record represents the main method for documenting the patient’s health encounter. The importance of documentation in a patient’s record has clearly been outlined in one of the provisions of the Saudi telemedicine regulations [30]. The regulation states that health care providers need to have access to the patient’s relevant health information and that all patient’s data and activities conducted during a telemedicine encounter be documented in the patient’s medical record [43]. A possible solution for this significant concern is incorporating the Shared E-Health File; a unified national electronic system that enables information exchange among different hospitals [44]. Incorporating an access to the Shared E-Health File within telemedicine apps [30] may potentially improve the level of care provided to patients and data interoperability. Specific measures would need to be put in place to overcome the challenges that the unified medical record system and the EHR cloud systems may bring. Challenges such as data protection and security issues are critical challenges for its acceptance among patients and healthcare providers [45,46].

Second, there was a slight difference between the usability issues identified during both evaluations based on the pandemic timeline. Although the number of usability issues were higher during our first evaluation, the average severity ratings for all apps were higher during our second evaluation. This may indicate the developers’ efforts in continuously working towards enhancing the users’ experience. In both evaluations, there were issues with “help and documentation”. Adding a separate accessible page outlining user instruction on how to use the app and access the telemedicine service is vital in enhancing the overall user experience [47]. Without having adequate user instructions, users may find difficulty in using the app, particularly with lack of technical support contact and the different types of users. When developing these apps, several age-related issues should be considered including cognition, perception, and behavior issues [8]. Providing help and support also is needed to overcome some technological barriers such as low technology literacy related to using telemedicine apps [48]. Although the apps we reviewed in our study were overall user friendly, special consideration should be provided to consider experienced and non-experienced users since we found “lack of flexibility” common within all three apps. Enabling users to customize user interfaces and create shortcuts might add a more personalized approach and a positive user experience [16].

Third, the rapid deployment of telemedicine apps in anticipation of a surge in COVID-19 cases may explain why we found most of the identified issues categorized as major problems and four out of seven catastrophic usability issues in “Dr. Sulaiman Alhabib” app’s user interfaces. “Dr. Sulaiman Alhabib” app’s telemedicine service was the only service developed in response to the pandemic and to changes to the country’s insurance policies [22]. It remains to be seen whether this service will last beyond the resolution of the pandemic and what role this will have on the use of telemedicine, particularly for their hospital’s patients

Lastly, the evaluation process itself resulted in identifying shared user tasks among the three apps. These tasks outlined the steps the user needed to perform to complete a specific telemedicine encounter. The identification of tasks helped us categorize usability issues into structured locations, which could potentially be used for future studies focusing on performing a cognitive walkthrough as a usability evaluation method [49].

Our study has several limitations. First, our app selection process was based on a publication issued by the NDT during the early months of the pandemic. These apps may not have represented the most used apps by the public during the time of our study. Relying on a different source, such as top downloads in App Store or Google Play, could have resulted in other apps included in our evaluation. Second, we conducted a heuristic evaluation, which depends on experts’ expertise. While this type of evaluation has proven useful in identifying usability issues, it may not be comprehensive in identifying all difficulties, which may be captured in usability tests with human participants [33]. Conducting a usability user test, which includes both types of users (healthcare providers and patients), considering different age groups may overall enhance the user experience. Lastly, because we used a heuristic evaluation method to assess the usability of the user interface, which is considered a method with limited generalizability [50], our study findings may be limited. Utilizing a combination of evaluation methods, such as cognitive walkthroughs and simulated interaction may provide a more comprehensive picture.

## 5. Conclusions

Heuristic evaluation studies have the potential to assist software designers and developers to discover severe usability issues that may have an effect on user acceptance of these apps. We evaluated three telemedicine apps used in Saudi Arabia using a heuristic evaluation method with a focus on understanding the usability issues in the apps user interface during COVID-19. We identified 54 user interface usability issues that may have an effect on the overall usability. Overall, our findings indicate that the three apps have a room for improvement by enhancing their user interfaces to improve the overall user experience.

## Figures and Tables

**Table 1:** Apps Features.

Feature	“Seha”		“Cura”		“Dr. Sulaiman Al Habib”	
	2020	2021	2020	2021	2020	2021
Ability to access educational information on COVID-19	√	√	√	×	×	×
Includes COVID-19 patient self-assessment tool	√	×	×	×	×	×
Limit to number of patient consultations	√	√	√	× ^+^	× ^+^	× ^+^
Patient able to choose among physician specialties	×	×	√	√	√	√
Patient able to see physician details	√	√	√	√	√	√
Supports video call	√	√	√	√	√	√
Supports text messaging	√	√	√	√	×	√
Supports voice messaging	√	√	√	√	×	×
Ability to attached and send files	√	√	√	√	×	×
Patient able to schedule a telemedicine consultation	√	√	√	√	×	√
Patient able to receive on demand consultation	√	√	√	√	√	√
Physician able to order a prescription	√	√	√	√	√	√
Linked to patient medical record	×	×	×	×	√	√
Patient able to view past consultation	√	√	√	√	√	√
End with satisfaction survey	√	√	√	√	√	√

+ Depends on each user’s insurance coverage plan.

## Data Availability

Not applicable.

## Appendix A

**Table A1:** List of mHealth apps published by NDT [29] in 2020 during the time of our study *.

No.	mHealth App	App Summary Description
1	Seha	Developed by the Ministry of Health (MOH) providing health and preventive care through audio-video medical consultations by MOH’s specialists, and through artificial intelligence technologies.
2	Mawid	Developed by the Ministry of Health (MOH), to enable patient to book their appointments across primary health care centers and manage them by canceling or rescheduling. As well as managing their referral appointments.
3	Asefni	Developed by the Saudi Red Crescent Authority providing ambulance emergency services in Saudi Arabia
4	Dr. Suliman Alhabib	Developed by Dr. Sulaiman Al Habib Medical Services Group providing access to patient portal services (such as managing medical records, checking lab results and radiology reports, booking appointments, checking prescriptions) and telemedicine care.
5	Cura	Developed by Ubieva providing healthcare services 24/7 from a distance.
6	Kingdom Hospital	Developed by the Kingdom Hospital providing patient portal services (such as managing medical records, checking lab results and radiology reports, booking appointments, checking prescriptions).
7	Web Teb	Developed by Web Teb proving health and medical news and accurate health information for consumers.
8	Nahdi	Developed by Al Nahdi Medical Company as a pharmacy app delivering pharmaceutical needs to customers.
9	Al dawaa Pharmacies	Developed by Al-Dawaa Medical Services Co as a pharmacy app delivering pharmaceutical needs to customers.
10	Mouwasat Medical Services	Developed by Mouwasat Medical Services providing services, which allow patients to book appointments, choose nearest hospital and required medical specialty.

* The list of apps included in the NTD document may have since been updated to include more apps.

## Appendix B

Form used for evaluation, which was filled out independently by each reviewer using Google forms.

*App Features*

1—Name of app:

- Seha
- Dr. Suliman Alhabib
- Cura

2—Type of patients the application serves

- Private
- Governmental

2—What clinical specialties are provided to the patient?

3—Is there a limit to the number of consultations with the healthcare provider per month?

- Yes
- No

4—Indicate the availability of the below features:

- COVID-19 screening information
- COVID-19 self-assessment tool
- Ability to choose a certain physician
- Ability to see physician details
- Text messaging
- Voice messaging
- Video call
- Ability to attach and send files
- Ability to schedule a tele-consultation
- Ability to receive on demand consultation
- Prescription
- Link to patient medical record
- Ability to view past consultation
- Satisfaction survey

*Usability Heuristics Evaluation Based on Nielsen’s Heuristics*

Please rate each usability heuristics item based on your inspection.

	**0—May Not Be a Problem**	**1—Cosmetic Problem Only**	**2—Minor Usability Problem**	**3—Major Usability Problem**	**4—Usability Catastrophe**
Visibility of system status					
Match between system and the real world					
User control and freedom					
Consistency and standards					
Error prevention					
Recognition rather than recall					
Flexibility and efficiency of use					
Esthetic and minimalist design					
Help users recognize, diagnose, and recover from errors					
Help and documentation					

## Appendix C

**Table A2:** “Seha” app (versions 1.0.35 and 1.0.36): usability issues identified based on Nielsen’s heuristics.

#	Heuristic Item	Evaluation Period	Usability Issue Description	Location	Rating	Redesign Recommendation
1	Visibility of system status	First (2020)	No identified			
		Second (2021)	The timer in the consultation room: unclear what it reflects. Does it reflect the user consultation time limit or the waiting time to see the physician?	During consultation	3	Add description of timer, i.e., waiting time to see physician or consultation duration.
			It took a while to load the consultation page to start consultation with physician.	Waiting for physician	1	Add a message indicating “loading”.
2	Match between system and the real world	First (2020)	User may not understand the meaning of “artificial intelligence” feature named “Smart Seha”	Log in	1	Add a definition of “Smart Seha” for the user in lay terms such as an “electronic tool that helps you understand your symptoms and recommends some actions”.
		Second (2021)	None identified			
3	User control and freedom	First (2020)	On the “consult physician” screen- when the user enters information, chooses “live session”, then chooses to cancel after seeing the waiting time, the app doesn’t go back to the previous screen “consult physician”, the app takes the user to the home screen.	Consultation initiation	3	Allow the app to take the user to the previous screen and not the home screen.
			† If the user screen goes static, the app does not give a notification to the user that a physician is present in the session and the app automatically ends the consultation without the option of going back to the session.	Waiting for physician	3	Allow the app to send a notification with sound to alert the user when a physician is present in the session and reply to the user.
		Second (2021)	The “back” icon in the consultation room takes the user to the home screen and not to the previous page (page where the user entered the consultation details). This happens without giving a notification where the back icon will take the user.	Consultation initiation	3	Change the icon of the icon to show a “home” icon rather than an arrow indication “back”—or program the app to go to the previous page instead of the home.
			The “back” icon and “end consultation” icon have the same functionality.	End of consultation	3	Differentiate between both icons by creating pages that reflect the functionality of the standard icon.
4	Consistency and adherence to standards	First (2020)	None identified			
		Second (2021)	Both “Smart Seha” and “health check” are artificial intelligence functionalities and it is unclear what the differences between these services are.	Log in	2	Add a description for each functionality in the home page.
			The term “health check” may also reflect a tele-consultation with a physician.	Log in	3	Add a description for each functionality in the home page.
5	Error prevention	First (2020)	† No notification indicating that if the user screen is static, the consultation will end and will be counted towards the user’s monthly consultation limit.	Waiting for physician	4	Create a notification for the user upon entering the chat room, which indicates that the consultation session will end if no response comes from the user.
		Second (2021)	When the user clicks on consultation by mistake, the app does not send a confirmation message to the user to start the consultation. This then counts as a consultation limit if the user decides to leave without seeing the physician.	Consultationinitiation	3	Allow the app to count active sessions (interaction between the physician and user)—as part of the monthly consultation limit and provide a follow-up on the experience of the consultation.
6	Recognition rather than recall	First (2020)	† Waiting time is only displayed before entering the consultation session room reflecting the time the user gets access to the room. When the user is in the room, waiting time for the physician to start the session is not displayed.	Waiting for physician	2	Provide a countdown timer within the consultation session screen showing the estimated waiting time for the physician to join.
			After the user leaves the open consultation session, the icon for reentering the consultation is not clear for the user.	During consultation	3	Add “open consultation” icon with visible instructions in every page.
		Second (2021)	When the user goes out of the consultation room by mistake, the app does not show a notification that “ you are in consultation”.	During consultation	3	Show a notification to user “you are in consultation”.
7	Flexibility and efficiency of use	No accelerators or ability to tailor frequent actions based on inexperienced and experienced users were found.				
8	Esthetic and minimalist design	First (2020)	Irrelative and unclear icons shown at the end after the “consultations page” to view the history of consultations indicating “closed”. This icon is “action required”.	End of consultation	1	Remove the “action required” icon from the closed consultations.
			There are two icons that lead to the same function “starting the telemedicine consultation”. One accessed in the home screen “consultations” and the other in consultations “new”, which brings the user back to the home screen.	Consultation initiation	1	Remove the “new” tab from the consultations screen. Main dashboard might provide a summary of the features offered on a high-level.
		Second (2021)	None identified			
9	Recognition diagnosis, and recovery from errors	First (2020)	None identified			
		Second (2021)	None identified			
10	Help and documentation	First (2020)	Quick start guide is only displayed to the users when the app is opened for the first time.	Log in	3	Provide users with ongoing access to help through an icon or tab placed in the chat room and/or in the home screen as user instructions.
		Second (2021)	The app does not provide clear directions on how to use the app, and what each icon or label means.	Orientation	4	Provide any extra information that would be useful to users, along with the label.
Total Issues identified from the two evaluations: 18						

† The issue was resolved—no longer identified in our second evaluation.

**Table A3:** “Cura” app (versions 1.8.9 and 2.0.0): usability issues identified based on Nielsen’s heuristics.

#	Heuristic Item	Evaluation Period	Usability Issue Description	Location	Rating	Redesign Recommendation
1	Visibility of system status	First (2020)	None identified			
		Second (2021)	None identified			
2	Match between system and the real world	First (2020)	User may not comprehend the meaning of “instant consultation” vs. “specialized consultation” and “find a doctor” vs. “instant consultation” vs. “specialized consultation” in a tele-consultation setting.	Consultation initiation	1	Help the user decide and select the option that fits their needs. For example, users start with “instant consultation” and from there they can be referred to a specialist if needed.
		Second (2021)	None identified			
3	User control and freedom	First (2020)	† No exit icon or skip from the instructions page when the user clicks the icon (i). The user must go through all the instructions.	Orientation	2	Provide a skip icon to end the help instructions.
		Second (2021)	None identified			
4	Consistency and adherence to standards	First (2020)	The search for “find a doctor” is not clear if the user is searching for the “specialized consultation” or the “instant consultation”.	Consultation initiation	3	Create separate search lists based on the user’s choice.
			On the technical support page, the license number of some staff indicates “000” or other numbers. This is unclear to the user if it is not applicable, or the license number is not updated.	Orientation	3	Avoid using “000” and clearly indicate if the license number does not apply to certain staff.
		Second (2021)	There are some pages displaying “instant consultation” and others displaying “specialized consultation”.	Consultationinitiation	2	Standardize the terms or add a description under each term to indicate the difference in service.
5	Error prevention	First (2020)	“Short brief about your case” indicates between brackets as (optional) when in fact it is mandatory to proceed to session payment.	Consultation initiation	3	Remove the word “optional”.
		Second (2021)	None identified			
6	Recognition rather than recall	First (2020)	† On the “search for doctor” screen” the user may not recall what each doctor specialty icon on the left panel represents and the user may need to click on each icon to read the labels presented on the right panel.	Consultation initiation	2	Help users select the doctor specialty based on symptoms or area of body in the main page instead of browsing all doctor specialties.
		Second (2021)	There is no specific icon that represents “help”. Help videos are displayed with other information under “find doctor” tab.	Orientation	3	Add an icon representing “help” where users can easily recall.
7	Flexibility and efficiency of use	No accelerators or ability to tailor frequent actions based on inexperienced and experienced users were found.				
8	Esthetic and minimalist design	First (2020)	The main “search for doctor” screen displays too much information in one screen i.e., name of doctor, picture, title, specialty, and rating.	Consultation initiation	1	The name of specialty may be removed since it is indicated under the main screen heading. Rating can also be removed as it is shown when the user clicks on a specific doctor.
		Second (2021)	There is a tab to “find a doctor” and there is also the same tab under “clinic”. “Find doctor” tab includes several irrelevant information	Consultation initiation Consultation initiation	3 4	Remove the additional tab which is under the clinic. Only include information relevant to “find doctor”
9	Recognition diagnosis, and recovery from errors	First (2020)	None identified			
		Second (2021)	None identified			
10	Help and documentation	First (2020)	The location of where the support and help are displayed in the app (part of the doctor list) may confuse the user. † The icon (i) representing help may be confused with general information about the app.	Orientation Orientation	1 2	It should be under a separate help icon. Change the icon (i) to “help”
		Second (2021)	Difficult to retrieve the help page when a consultation with the physician is ongoing.	During consultation	4	Add a clear separate page with a help icon, which can be accessible.
Total Issues identified from the two evaluations: 14						

† The issue was resolved—no longer identified in our second evaluation.

**Table A4:** “Dr. Sulaiman Alhabib” app (versions 4.2.3 and 4.4.4): usability issues identified based on Nielsen’s heuristics.

#	Heuristic Item	Evaluation Period	Usability Issue Description	Location	Rating	Redesign Recommendation
1	Visibility of system status	First (2020)	The app does not provide enough feedback after pressing the start consultation button.	Consultation initiation	1	Provide constructive feedback describing what the system is doing.
		Second (2021)	Loading time was long. Does not indicate what the app is doing from one page to another, just shows the hospital logo.	Consultation initiation Orientation	1 2	Add a message indicating “loading”. Provide constructive feedback describing what the system is doing.
2	Match between system and the real world	First (2020)	When registering as a new patient, the app mandates the name in English.	Registration	2	Allow the user to choose the name in Arabic or English.
		Second (2021)	None identified			
3	User control and freedom	First (2020)	In the payment screen, when user wants to change method of payment, the back icon takes the user to the home screen to start over and not back to the payment method options. † The session starts a video directly without the patient’s consent.	Consultation initiation During consultation	2 4	Allow the user to go back to the previous payment method screen instead of the home screen. Notify the patient that the session will start in video or start with a voice call and then with video after patient approval.
		Second (2021)	The app allows the users to search the schedules of physicians before logging in. After the user has chosen a time, the app displays a message to the user to logs in. When the user login, the app returns the user to the home screen to search again.	Consultation initiation	2	Ask the user to log in before searching the schedules of physicians or prevent the app from going back to the first step (home screen) after the user logs in.
4	Consistency and adherence to standards	First (2020)	† How to access the telemedicine service from the home page is unclear to the user, i.e., what is the difference between the live care icon and the request appointment icon.	Consultation initiation	2	Make the live care icon more visible to the user by creating an option to choose from a list of consultation types, e.g., telemedicine-live, or physical visit by appointment
		Second (2021)	After selecting” live care’’, there are two tabs: “consultation” and “name of the doctor”, which confuse the user.	Consultation initiation	3	Add a description under each tab
5	Error prevention	First (2020)	When registering as a new patient, the app does not indicate the name in English as a requirement. † No confirmation message for the user to end the session.	Registration End of consultation	2 2	Inform the user or provide an early error message when Arabic letters are written. Show a confirmation message before ending the session.
		Second (2021)	The app did not provide an error prevention message during log in stage. In general, the app did not provide any error prevention messages.	Log in Registration	4 4	Add notification messages throughout the app, indicating and error will occur if the user proceeds or clicks a certain tab or icon.
6	Recognition rather than recall	First (2020)	† No instructions on how to use the app.	During consultation	4	Provide the user with clear instruction before the start of the live care session.
		Second (2021)	None identified			
7	Flexibility and efficiency of use	No accelerators or ability to tailor frequent actions based on inexperienced and experienced users were found.				
8	Esthetic and minimalist design	First (2020)	The home page dashboard has so many displayed icons, which may confuse the user. The live session screen has icons placed in the bottom panel that may not be needed during the live consultation session such as “book appointment” and “actions”.	Registration Consultation initiation	2 1	Design a more minimalist and esthetic home screen. Remove “book appointment” and “actions” icons from the live session screen.
		Second (2021)	“My Medical File” tab has many icons, which may confuse the user, when accessing records.	Registration	1	Minimize the icons and information displayed, by allowing the patient to personalize the page.
9	Recognition diagnosis, and recovery from errors	First (2020)	The error presented to the user because of choosing an unavailable clinic does not show a recovery message that the clinic has ended. The user must close the application and start over.	Consultation initiation	1	Add real-time updates indicating the available clinics (currently online) vs. unavailable clinics (offline) and provide a message to the user indicating what to do in case he/she chooses an unavailable clinic.
		Second (2021)	The error presented to the user was not clear and did not explain the error and the solution.	Consultation initiation	2	Add clear error messages associated with clear instructions on how to resolve the error
10	Help and documentation	First (2020)	Instructions on how to use the “live care” feature are not presented to the user with no technical support contact information in case the user needs assistance.	Orientation	4	Provide users with easy access to instructions on how to use the app and on the home screen as user instructions. Provide contacts for help and support or live chat for technical issues.
		Second (2021)	Instructions on how to access the help giving instructions on how to use the “live care” feature are not clear to the user. Voice recognition help is not appropriately working.	Orientation during consultation	2 4	Add a clear separate page with a help icon, explaining how the app is used. Improve or remove the feature.
Total Issues identified from the two evaluations: 22						

† The issue was resolved—no longer identified in our second evaluation.
